# Improved Acuity and Dexterity but Unchanged Touch and Pain Thresholds following Repetitive Sensory Stimulation of the Fingers

**DOI:** 10.1155/2012/974504

**Published:** 2012-01-18

**Authors:** Rebecca Kowalewski, Jan-Christoph Kattenstroth, Tobias Kalisch, Hubert R. Dinse

**Affiliations:** ^1^Neural Plasticity Laboratory, Institute for Neuroinformatics, Ruhr-University Bochum, 44780 Bochum, Germany; ^2^Department of Neurology, Ruhr-University Bochum, BG-Kliniken Bergmannsheil, 44789 Bochum, Germany

## Abstract

Neuroplasticity underlies the brain's ability to alter perception and behavior through training, practice, or simply exposure to sensory stimulation. Improvement of tactile discrimination has been repeatedly demonstrated after repetitive sensory stimulation (rSS) of the fingers; however, it remains unknown if such protocols also affect hand dexterity or pain thresholds. We therefore stimulated the thumb and index finger of young adults to investigate, besides testing tactile discrimination, the impact of rSS on dexterity, pain, and touch thresholds. We observed an improvement in the pegboard task where subjects used the thumb and index finger only. Accordingly, stimulating 2 fingers simultaneously potentiates the efficacy of rSS. In fact, we observed a higher gain of discrimination performance as compared to a single-finger rSS. In contrast, pain and touch thresholds remained unaffected. Our data suggest that selecting particular fingers modulates the efficacy of rSS, thereby affecting processes controlling sensorimotor integration.

## 1. Introduction

Adult mammalian brains maintain plastic reorganizational capacities throughout life that mediate learning processes [1–3]. In particular, neural connections and connection strengths are modified during extensive use, practice, and training. For example, Braille readers exhibit an enlarged cortical representation of the reading finger in S1 [4], and the cortical representations of the fingers of the left hand are increased in string players [5].

Over the years, it became clear that, in addition to training, practice, and perception, behavior and cognition can be systematically improved in human subjects simply through exposure to sensory stimulation. These stimulation paradigms are characterized by the fact that they employ the timing conditions of canonical protocols used to alter synaptic transmission and efficacy [6–11]. Based on the framework of Hebbian synaptic plasticity, we developed a specific stimulation paradigm (coactivation) that influences brain activity specifically. The idea behind coactivation is the simultaneous activation of mechanoreceptors of the skin or of the peripheral nerve fibers [7]. The stimulation paradigm was applied by small devices consisting of a solenoid that was taped to the tip of the index finger for a few hours to induce synchronous neural activity by tactile costimulation of small skin portions. As a result of this unattended activation-based learning, the tactile acuity of the stimulated body part improved parallel to an enlargement in the respective cortical representation [12–14]. Recently, we developed alternative protocols that focus on frequency rather than spatial cooperative processes by using high-frequency stimulation [8], which presumably induce long-term potentiation-like (LTP-like) processes in the brain. This form of repetitive sensory stimulation (rSS) was further optimized and modified by reducing the duration of application from a few hours to 20 min [8] and by using not only cutaneous but also electrical stimulation, where electrical pulses were transmitted by self-adhesive electrodes. Currently, different forms of rSS procedures are widely investigated by different groups as a means to drive learning and plasticity processes by using different terms such as “peripheral nerve stimulation” [15], “exposure-based learning” [16], “coactivation” [13, 14, 17], “unattended activation-based learning” [7], and “rSS” [18]. By adopting protocols consisting of intermittent high-frequency or low-frequency stimulation for the visual domain, we were recently able to demonstrate a substantial modifiability of visual perception and behavior in human individuals indicating a similar readiness for passive stimulation-induced changes as shown thus far in the somatosensory system [6].

The sense of touch is not a uniform entity, but comprises quite diverse features. From an operational point of view, investigation of the sense of touch requires breaking down performance and functions related to touch into measurable variables. In our studies on the plasticity of the sense of touch, we have referred to a hierarchy of tasks and task complexities, which differ in the involvement of proprioception and motor functions, as well as the amount of cognitive demand [19]. Accordingly, the underlying neural substrates differentially involve, in a graded way, contribution from the periphery and from various cortical areas, including primary, input-receiving areas as well as higher-order, associative, and often multimodal areas. Previous studies employing EEG, MEG, and recording of BOLD signals have shown that rSS modulates activation in primary and secondary somatosensory cortices [12–14, 20, 21]. In fact, animal studies had indicated that receptive fields and cortical maps in the paw representation of somatosensory cortex are modified by rSS protocols [22].

In the present study we therefore investigated the impact of repetitive sensory stimulation (rSS) not only on tactile discrimination performance, but additionally on dexterity, touch sensitivity, and the perception of pain to further explore the potential of rSS in evoking beneficial effects on tactile sensation and perception and sensorimotor performance beyond acuity. Our main hypothesis was that rSS might affect some aspects of the diverse features of the sense of touch, but not all.

Tactile spatial discrimination performance of the fingers can be modulated by applying rTMS above the finger representation of SI implying a contributing role of SI [23], although other, putative multisensory areas appear to be involved in human acuity processing [24]. Dexterity of the hand and fingers was investigated using the pegboard test, which characterizes the abilities of sensorimotor integration during precision grip. Since precision grip is typically performed with thumb and index finger, we applied rSS to those two fingers in order to maximize possible effects on dexterity. Sensorimotor integration is based on feedforward and feedback contributions between different cortical areas including Brodmann's areas 1, 2, 3a, 3b, 5, and 7 and the motor areas including Brodmann's areas 4 and 8 [25–29]. In addition, a number of human brain mapping studies have described activation of somatosensory cortex after the execution of a motor task. In monkeys, tactual-motor skill training resulted in large-scale reorganization of area 3b [30]. Accordingly, the joint activation of both cortical regions supports the idea of a profound interconnectedness in the sensorimotor system. We thus hypothesize that rSS affects directly the somatosensory pathway, and additionally modulates the exchange of information between the somatosensory and motor system, resulting in an improvement of fine manipulative abilities.

The perception and detection of nonpainful stimuli is typically characterized by measuring touch thresholds. Conceivably, SI as the main receiving input station can be assumed to play a role in processing of mechanically and electrically generated tactile inputs [31]. Earlier studies using single pulse transcranial magnetic stimulation (TMS) have shown that application of TMS above SI attenuates the detection of an electrical stimulus to the index finger [32]. Interestingly, detection of somatosensory stimuli can also be reduced after application of TMS above parietal areas indicating a network of areas [33].

Transcutaneous electrical nerve stimulation (TENS) is often used as a means to attenuate pain. It has been suggested that the pain-relieving action of TENS is in part due to a release of endogenous opioids [34, 35]. On the other hand, imaging studies have indicated that somatosensory areas near the lateral sulcus (Sylvian fissure) are implicated in pain processing. In addition, there is evidence for additional representations of pain in the deep parietal operculum and anterior insula, and secondary somatosensory cortex [36]. We therefore decided to test whether rSS, which affects SI and SII [14], also imposes beneficial effects on the nociceptive system.

Our data show that simultaneously exposing 2 fingers to rSS improves dexterity and potentiates the effects on tactile acuity without affecting touch and pain thresholds.

## 2. Methods

### 2.1. Subjects

We tested a total of 26 right-handed subjects (mean age: 23.62 ± 2.38 years, 13 females). All subjects gave their written informed consent, and the protocol was approved by the local ethics committee of the Ruhr-University Bochum. The protocol was performed in accordance with the Declaration of Helsinki.

### 2.2. Assessment of 2-Point Discrimination Threshold

The 2-point discrimination (2pd) threshold is a reliable marker of tactile acuity in humans. The 2pd thresholds were assessed on the tips of the thumb (*d1*), index finger (*d2*), and ring finger (*d4*) of the right hand by using the method of constant stimuli described previously [7, 9, 12–14, 17, 37]. A custom-made device was used to assess the 2pd thresholds on a fixed position on the skin of the fingertips by rapidly switching between stimuli. The stimuli consisted of 7 pairs of brass needles with different distances (ranging from 0.7 to 2.5 mm in increments of 0.3 mm) and a single needle as 0 distance (control condition). The needles were 0.7 mm in diameter with blunt ends that were approximately 200 *μ*m in diameter. Tactile stimuli were applied for approximately 1 s; application forces were 150 to 200 mN. The subjects were instructed to place their finger on the support and to maintain the initial position of the finger. The stimuli were presented 10 times in randomized order resulting in 80 trials per session. Subjects were not informed about the ratio of needle pairs and single needles, which was 7 : 1. Subjects had to decide immediately after stimulus application if they had the sensation of 1 or 2 needles by reporting the percept of a single needle or of doubtful stimulus as “1,” but the distinct percept of 2 stimuli as “2.” All responses were plotted against needle distances resulting in a psychometric function, which was fitted by a binary logistic regression. The 2pd threshold was taken from the fit where 50% correct responses were reached. All subjects had to accomplish 1 training session to become familiar with the testing procedure.

### 2.3. Assessment of Touch Threshold

Touch thresholds were assessed by probing the fingertips of the thumb (*d1*), index finger (*d2*), and ring finger (*d4*) of the right hand with von Frey filaments (Marstocknervtest, Marburg, Germany), following the procedures described with Semmes-Weinstein monofilaments [38, 39]. Each filament was calibrated to a known buckling force determined by its length and diameter. The test kit consisted of 16 different filaments with forces ranging from 0.25 mN to 10 mN in logarithmic scaling. Additionally, 2 filaments with forces of 0.08 mN and 0.20 mN were used to expand the test range (Touch Test, Stoelting Co., Wood Dale, IL, USA). Touch sensitivity was investigated by using a staircase procedure during which subjects were required to close their eyes and report when they perceived an indentation of the skin on their fingertips. The applied forces, starting with a noticeable stimulus, were decreased in a stepwise manner until the subjects no longer perceived the stimulus (lower boundary) and then increased until the stimulus was perceived again (upper boundary). This procedure was repeated 3 times resulting in 6 values that were averaged to provide the touch threshold.

### 2.4. Assessment of Pressure Pain Threshold

The pressure-pain threshold (PPT) is defined as the minimum force necessary to cause a painful sensation [40, 41]. For PPT measurement on the tips of *d1*, *d2,* and *d4* of the right hand, a Force-Dial FDN 200 algometer was used (Wagner Instruments, Greenwich, CT, USA). It contains a plastic housing and a stainless steel plunger with a diameter of 6.5 mm, which was positioned on the tip of the tested finger. Then, the algometer was pressed down slowly by the experimenter who immediately stopped the measurement when the subject reported a painful sensation. The force required to induce pressure pain was expressed in Newton (N).

### 2.5. Pegboard Test

The pegboard test investigates fine and gross motor dexterity and coordination of hands, fingers, and arms [42]. To the right side of the subject, a 5 × 30 cm ledge with 25 drilled holes was located. A container with 25 metal pins was placed 30 cm from the ledge. The subjects were instructed to pick the pins with their right hand one by one from the container and to insert them into the holes on the ledge. If one of the metal pins dropped during performance, subjects were instructed to go on with the next pin. Performance was measured using the time to complete the test and the number of dropped pins. The test was performed in a standard version (size of metal pins 5 ∗ 0.25 cm) and in a more demanding version with smaller pins (size of metal pins 1 ∗ 0.25 cm). Subjects were instructed to fulfill the task as fast as possible. To establish a stable baseline performance before application of rSS, subjects had to perform the test 3 times. After rSS, the test was repeated once to evaluate performance in the *post-*condition.

### 2.6. Electrical rSS

Repetitive sensory stimulation was applied for 30 min on *d1* and *d2*. The rSS sequence consisted of stimulus trains of 1 s (single-pulse duration: 0.2 ms [square], frequency: 20 Hz) and intertrain intervals of 5 s [8]. The sequence was played back from a digital storage that triggered a standard TENS device (Pierenkemper, Germany). The pulses were transmitted by adhesive surface electrodes (1 ∗ 4 cm, Pierenkemper, Germany) fixed on the first and third segment of each finger (cathode proximal). Stimulation intensity was adjusted individually for each subject. Mean intensity was 1.38 ± 0.14 mA.

### 2.7. Experimental Schedule

All tests described were conducted before (*pre*) and after (*post*) the application of rSS. The measurements of tactile and fine motor performance were assessed in all of the 26 subjects. Thresholds were assessed on *d1* and *d2* of the right hand and, additionally, on the tip of *d4* in a subgroup of 10 participants.

### 2.8. Statistical Analyses

Statistical analyses were done with IBM SPSS Statistics 19.0 for Windows. We used repeated measures (rm) ANOVA with SESSION as inner-subject factor to indicate differences between performances in the precondition. Single-session data were compared by means of post hoc test (Fisher LSD). To evaluate changes between *pre-* and *post-*sessions, the performances were compared by means of 2-tailed *t*-tests. Linear correlation analyses were calculated by means of 2-sided Pearson correlations. All results are presented as means ± standard error of mean in the text. The *P* values ≤ 0.05 were considered significant.

## 3. Results

### 3.1. Two-Point Discrimination Threshold

**Figure 1:**
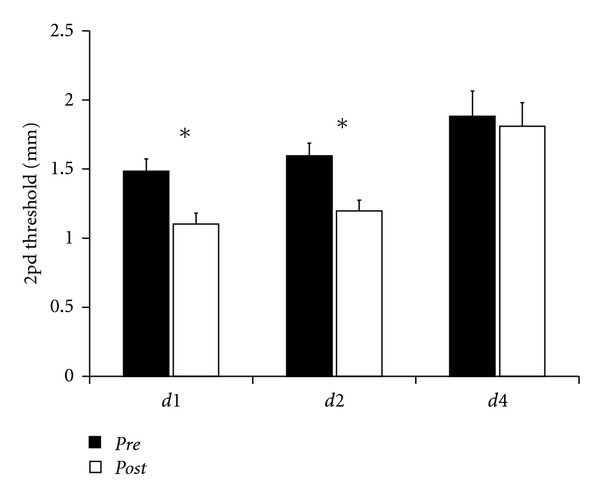
Spatial 2pd thresholds of *d1*, *d2* (*n* = 26), and *d4* (*n* = 10). After rSS, the thresholds of *d1* and *d2* were significantly decreased, while 2pd thresholds of *d4* did not change. Stars indicate significant differences (*P* ≤ 0.05) to the precondition.

Average 2pd thresholds were calculated for the thumb (*d1*), index finger (*d2*), and ring finger (*d4*) (Figure 1). To obtain a stable baseline of discrimination, we tested the subjects' performance with the right index finger in 2 consecutive sessions. We found discrimination thresholds of 1.65 ± 0.09 mm in session 1 and 1.60 ± 0.09 mm in session 2, which were not significantly different (rmANOVA; *F*_(1,25)_ = 3.032; *P* = 0.094; test-retest reliability: Cronbach's *α* = 0.917). In accordance with previous studies, data obtained during session 2 were used as *pre*-values. For the other fingers under precondition, we found discrimination thresholds of 1.49 ± 0.09 mm for *d1* and 1.88 ± 0.18 mm for *d4*.

 After the application of rSS, the thresholds of *d1* and *d2* were significantly lowered to 1.10 ± 0.08 mm and 1.20 ± 0.08 mm, respectively, (*t*-test; *P* ≤ 0.001) which corresponds to an average percentage improvement of 26%. On the contrary, the 2pd threshold of the not stimulated *d4* did not change (*post *1.81 ± 0.17 mm; *t*-test; *P* = 0.256).

### 3.2. Touch Threshold

**Figure 2:**
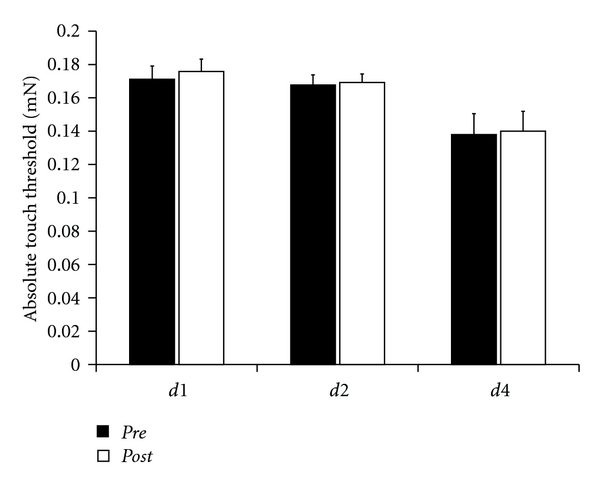
Touch thresholds of *d1*, *d2* (*n* = 26), and *d4* (*n* = 10). There were no significant alterations of thresholds after rSS (*P* ≥ 0.261).

Touch thresholds were 0.17 ± 0.01 mN for *d1*, 0.17 ± 0.01 mN for *d2,* and 0.14 ± 0.01 mN for *d4*. In the *post-*session, we assessed values of 0.18 ± 0.01 mN for *d1*, 0.17 ± 0.01 mN for *d2,* and 0.14 ± 0.01 mN for *d4* (Figure 2). The differences were not significant for any finger (*t*-test; *P* = 0.261 [*d1*]; *P* = 0.574 [*d2*]; and *P* = 0.678 [*d4*]).

### 3.3. Pressure-Pain Threshold

**Figure 3:**
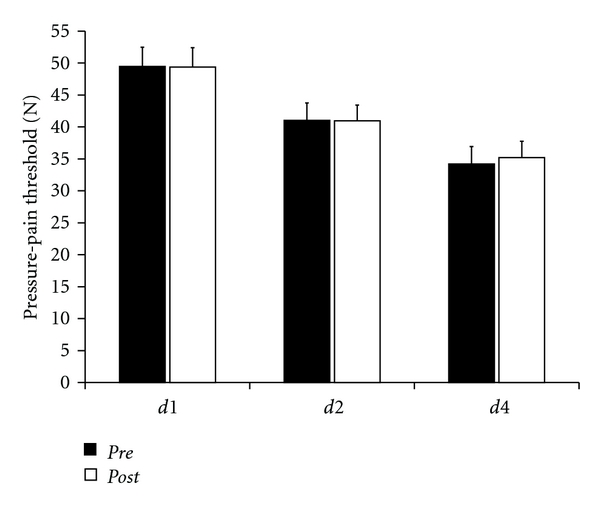
Pressure-pain thresholds of *d1*, *d2* (*n* = 26), and *d4* (*n* = 10). There were no significant changes after rSS, neither for the stimulated fingers (*d1* and *d2*; *P* ≥ 0.896), nor for the not stimulated finger (*d4*; *P* = 0.213).

Average PPTs were 46.46 ± 3.03 N for *d1*, 41.04 ± 2.72 N for *d2*, and 34.20 ± 2.74 N for *d4* in the *pre-*session (Figure 3). After rSS, we found pain thresholds of 49.38 ± 3.03 N for *d1*, 40.96 ± 2.47 N for *d2,* and 35.20 ± 2.57 N for *d4*. None of the differences were significant (*P* = 0.896 [*d1*]; *P* = 0.952 [*d2*]; *P* = 0.213 [*d4*]).

### 3.4. Pegboard Test

**Figure 4:**
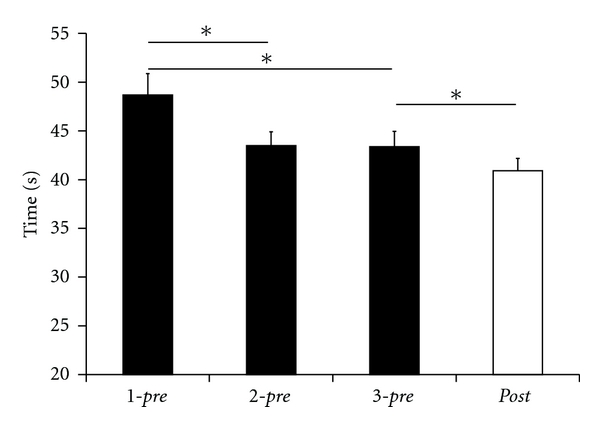
Time to complete the pegboard test in the demanding version (short pins). Subjects reached a stable baseline after completing the test once. After rSS following session 3, subjects needed significantly shorter times to complete the task (session 4, *post-*condition). Stars indicate significant differences (*P* ≤ 0.05).

We evaluated pegboard performance by measuring the time to complete the test and the number of errors for the standard (long pins) and for a more demanding version (short pins). Before application of rSS, the subjects had to complete both versions of the tests 3 times to obtain a stable baseline of performance. For both versions, the subjects showed task improvement between the first and second, but not between the second and third session, indicating that subjects had reached a stable plateau of performance (Figure 4). The average time for completion in the demanding version (short pins) was 48.69 ± 2.18 s in *1-pre*, 43.50 ± 1.41 s in *2-pre*, and 43.38 ± 1.56 s in *3-pre*. The rmANOVA for factor SESSION revealed significant differences of time (*F*_(2,50)_ = 9.190; *P* ≤ 0.001) for the 3 *pre*-sessions. Subsequent post hoc analysis (Fisher LSD) revealed a significant reduction of time from *1-pre* to *2-pre* (*P* = 0.039), from *1-pre* to *3-pre* (*P* = 0.035), but not from *2-pre* to *3-pre* (*P* = 0.936).

**Figure 5:**
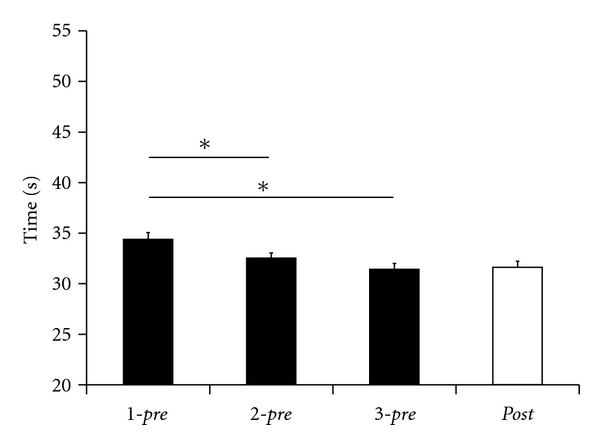
Time to complete the pegboard test in the standard version (long pins). Subjects reached a stable baseline after completing the test once. After rSS following session 3, subjects did not change their performance significantly (*P* = 0.632). Stars indicate significant differences (*P* ≤ 0.05).

A similar pattern of behavior was found in the standard version of the pegboard test (long pins). In general, subjects needed less time to complete this test version. The average time to complete the task was 34.38 ± 0.67 s in *1-pre*, 32.54 ± 0.50 s in *2-pre,* and 31.42 ± 0.58 s in *3-pre* (Figure 5). The rmANOVA for factor SESSION indicated significant changes in performances in the single sessions (*F*_(2,50)_ = 34.216; *P* ≤ 0.001). Post hoc analysis (Fisher LSD) showed a significant shortening of time from *1-pre* to *2-pre* (*P* = 0.029), from *1-pre* to *3-pre* (*P* = 0.001), but not from *2-pre* to *3-pre* (*P* = 0.182).

While after rSS the subjects' performance improved significantly in the demanding version, there were no significant improvements in the standard version. The time needed to complete the test using the small pins significantly reduced from 43.38 ± 1.56 s in *3-pre* to 40.92 ± 1.26 s in the *post-*session (*t*-test; *P* = 0.032).

The time needed to complete the test using the long pins changed from 31.42 ± 0.58 s (*3-pre*) to 31.62 ± 0.61 s in the *post-*session (*t*-test; *P* = 0.632).

The evaluation of the parameter number of errors revealed that the subjects made more mistakes when performing the demanding version. On average, in the standard version of the pegboard test they made 0.31 ± 0.13 errors in *1-pre*, 0.31 ± 0.12 errors in *2-pre,* and 0.04 ± 0.04 in* 3-pre*. Completing the task in the demanding version, the number of errors was 2.38 ± 0.47 in *1-pre*, 1.50 ± 0.47 in* 2-pre,* and 1.65 ± 0.34 in *3-pre*. However, the total number of errors made while completing the test in both versions was too small to indicate significant differences between sessions. As a result, the rmANOVA for factor SESSION revealed no significant differences for number of errors made in the *pre-*sessions 1 to 3, neither for the standard (*F*_(2,50)_ = 2.601; *P* = 0.084) nor for the demanding test version (*F*_(2,50)_ = 2.631; *P* = 0.082). After rSS, the subjects' error rates did not change. Average number of errors was 0.15 ± 0.07 in the standard version (*t*-test; *P* = 0.083) and 1.54 ± 0.34 (*t*-test; *P* = 0.780) in the demanding version.

### 3.5. Gain of Performance in Tactile Discrimination and Fine Motor Performance

**Figure 6:**
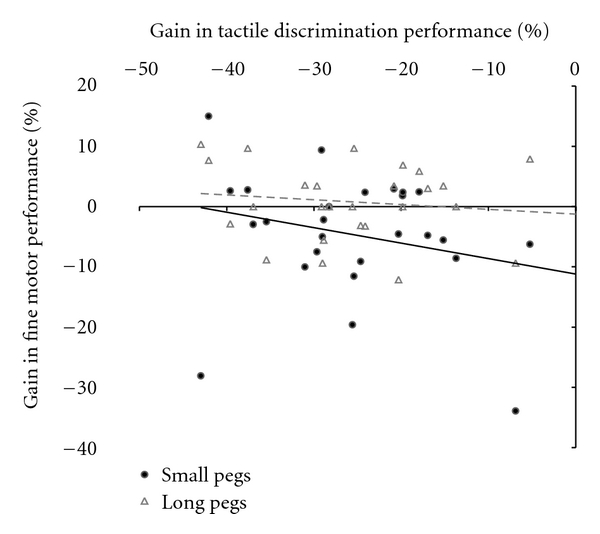
Gains in tactile discrimination and fine motor performance were not significantly correlated, neither for the demanding (*r* = −0.245; *P* = 0.227) nor for the standard version of the test (*r* = −0.098; *P* = 0.635). Gains of performance refer to percent changes from *pre-* to *post-*session.

Of the tested parameters, 2pd and dexterity were affected by rSS. We therefore investigated potential relationships between the gains in tactile discrimination (individual gains of *d1* and *d2* were averaged) and fine motor performance (gain of performance refers to percent changes from *3-pre* to *post*) by calculating Pearson correlation coefficients. We found no significant correlations between the gains in performance in 2pd and in the pegboard test in the standard version (*r* = −0.098; *P* = 0.635) or in the demanding version (*r* = −0.245; *P* = 0.227) (Figure 6).

## 4. Discussion

The application of repetitive electrical sensory stimulation simultaneously on 2 fingers of the right hand resulted in differential effects on perception, sensorimotor behavior, and touch or pain thresholds. While tactile spatial discrimination and dexterity improved, thresholds for touch or pain remained unaltered. Remarkably, the simultaneous stimulation of 2 fingers appeared to potentiate the beneficial effects previously described following the stimulation of a single finger.

Under baseline conditions, average 2pd thresholds of the thumb, index, and ring fingers of the right hand showed an increase in thresholds from *d1* to *d4*. A similar gradient of tactile acuity across fingers has been observed in previous studies [39, 43–45]. To account for this phenomenon, it has been suggested that precision grip is more often performed with the thumb and index finger than with the thumb and ring finger. Therefore, the thumb and index finger—which show the lowest thresholds—are permanently involved in movements of everyday life, for example, grasping or writing. The thumb, especially, plays an important role in holding, moving, and grasping objects because of its location opposite to the remaining fingers [45]. However, the differential frequency of use does not seem to be the only factor that determines spatial acuity. Other factors such as innervation density or the size of respective cortical areas might contribute to the gradient of spatial acuity [46–48].

After application of rSS to the thumb and index finger, the 2pd thresholds were significantly reduced. Earlier studies have shown that repetitive sensory stimulation of the form used here causes an improvement in tactile discrimination abilities in adult and aged individuals [8, 49].

Previously used stimulation protocols, for example, the tactile coactivation protocol, evoked an average improvement of tactile acuity of about 15% [13] when applied to a single finger. When coactivation was applied simultaneously to all fingers, a gain of approximately 20% was reported [9]. Using a high frequency stimulation protocol led to an improvement of about 16% [8]. In our present study, where we applied high-frequency stimulation to the thumb and the index finger, we observed an extralarge gain in tactile performance in the range of 26%, which implies synergistic effects. When the coactivation protocol was applied in a rat model of somatosensory plasticity, simultaneous coactivation on 2 neighboring digits resulted in an expansion of the corresponding cortical representations of the stimulated skin sites characterized by a fusion of both stimulated territories [22]. In another study, monkeys received temporally coincident inputs across fingertips and finger bases, but distal versus proximal digit segments were non-coincidentally stimulated. Electrophysiological recordings in the somatosensory cortex showed that synchronously applied stimuli resulted in integration of inputs in the cortical maps, whereas stimuli applied asynchronously were segregated [50]. Accordingly, simultaneous application of repetitive stimulation protocols appears to potentiate the positive effects observed following the stimulation of a single finger. This view is in line with recent studies on human tactile perception, which revealed major modulating effects of conditioning stimulation [51], in particular, when applied synchronously [52].

Hand and finger dexterity are regarded as a marker of sensorimotor integration abilities. Furthermore, tactile information is crucial for accurate motor control in fine manipulative tasks such as precision grips. We used 2 test versions that differed in task difficulty. This was corroborated by the fact that subjects needed more time to complete the test using small pins as compared to the test using long pins. Accordingly, the demanding test version required more resources for coordination of tactile, visual, and motor information. For both test versions, we found that after completing the first session, the subjects reached a stable baseline in their performance indicated by the observation that the performances in the second and third session were similar (Figures 4 and 5).

For this task, the thumb and index finger were exclusively used for grasping and holding the pins. It is therefore conceivable to assume that rSS applied to these two fingers contributed to, besides improving tactile acuity, an enhancement of dexterity and sensorimotor integration abilities. In fact, after the application of rSS, we observed a significant improvement of fine motor performance for the demanding test using small pins, but not for the version using long pins, which most likely is due to a ceiling effect, as the time needed to complete this test version was much shorter making it more difficult to detect small changes. The same may be true for the parameter number of dropped pins that did not reveal any rSS-induced alterations for both test versions.

A possible explanation for the transfer of beneficial effects from sensory stimulation to sensorimotor behavior is the interconnectedness of the somatosensory cortex with motor, premotor, and parietal cortices [53–56]. These interconnections elicit a cortical reorganization in the primary motor cortex after stimulation, resulting in increased excitability of the motor cortical representations [57, 58], in intracortical facilitation [59], and in a decrease in intracortical inhibition [60]. It has been suggested that these processes are modulated by GABAergic neurotransmission [61]. According to a functional magnetic resonance imaging (fMRI) study, the representation of the thumb in S1 significantly increased after a sensory stimulation of the medianus nerve, which was taken as an indication of the influence of somatosensory stimulation on motor cortical function, possibly supporting beneficial effects on motor control [62].

Another possibility is that the rSS-induced improvement of tactile acuity enhances tactile components that contribute to fine motor performance and execution. However, the correlation analysis between the gain of tactile discrimination performances and digital dexterity revealed no such relation. The complete lack of correlation between both parameters is in line with previous studies in elderly subjects [37] and with a recent report that studied the relation between acuity and dexterity during childhood [63]. It should be noted, however, that in elderly participants a close relationship between spatial acuity and dexterity had been observed [64]. Further support for a complex relation between dexterity and acuity comes from studies in patients suffering from median nerve compression, which revealed impaired acuity but normal pegboard performance [65].

The touch thresholds we measured were highest at the thumb and index finger, and lowest at the ring finger, corroborating a well-documented observation about a thumb to little finger gradient, which is opposite to the gradient of acuity [9, 37, 66–68]. This gradient most likely arises as the result of different mechanoreceptor densities across fingers [69–71]. Furthermore, it is possible that the contrasting behavior of tactile acuity and fine touch sensitivity across the fingers of a hand is due to differences in skin structure caused by differential use. Because of its opposing location, the thumb is exposed to higher mechanical forces more frequently than the ring finger, so changes in the skin structure prevent the perception of minimal mechanical loads. The differences in skin structure can be compensated for in the 2pd task insofar as the subjects are able to regulate the contact intensity between the skin and the mechanical stimuli [9].

In contrast to acuity and dexterity, touch thresholds were not significantly affected by rSS—an observation that has been already reported [9]. As we did not even observe touch threshold changes in elderly subjects who already have significantly higher thresholds, which would permit space for improvement, a ceiling effect appeared unlikely [37]. It had been argued that the beneficial effects of rSS result from changes in synaptic efficacy and synaptic connections. In contrast, touch thresholds seem to reflect predominantly peripheral factors such as mechanoreceptor density and mechanoreceptor composition, which remain unaffected by cortical plasticity processes. However, other attempts that interfere with sensory peripheral transmission have been described to successfully alter touch thresholds. For example, adding noise to a transmitted signal can improve the ability to reliably transfer information, a phenomenon known as stochastic resonance. Electrical noise stimulation applied to the hand lowered the touch thresholds of elderly individuals [72].

While it has been repeatedly shown that touch thresholds remain unaffected by rSS in adult and elderly individuals, we recently observed that application of rSS in patients suffering from subacute or chronic stroke elicits significant improvement of touch thresholds [73, 74]. We therefore suggested that rSS-induced improvement of touch thresholds can emerge under conditions where the processing of touch information is severely compromised as is the case in stroke patients.

Similar to touch thresholds, PPTs were not equally distributed across the fingers of the hand, but decreased from the thumb to the little finger. This observation is in line with earlier reports describing that PPTs of the index finger were higher than PPTs of the little finger [75].

The present study was the first where we addressed the question whether PPTs were influenced by rSS in young and healthy subjects. Our data showed very clearly that this was not the case. These experiments were motivated by the frequent use of TENS to ease the pain in patients suffering from chronic pain. TENS is assumed to trigger an opioid-mediated suppression of dorsal horn neurons through the concerted activation of the periaqueductal gray and the rostral ventral medulla [76]. For example, the concentrations of *β*-endorphins have been shown to increase in the bloodstream and cerebrospinal fluid of healthy subjects after administration of either high (101–108 Hz) or low (4–7 Hz) frequency TENS [77, 78]. The application of various TENS protocols in adult, healthy subjects lead to a significant increase of PPTs, with continuous high-frequency stimulations (80 Hz) being more effective in increasing PPTs [79]. However, there is evidence that high-frequency, high-intensity stimulation produced significant analgesic effects mainly during the stimulation period with little maintenance of the efficacy for only 20 min after the termination of TENS [80]. Accordingly, the lack of rSS effects on pain perception observed in our study may be attributable to several factors. It is possible that potential effects were too small to be assessed by our methods. Similarly, at the time point of postassessment, the possible effects might have recovered already. Another explanation is that pain is not primarily processed in somatosensory cortex, which is the main target area affected by rSS [12–14, 21]. Finally, the stimulation protocol used in our study differs from the typical TENS protocol and might therefore have failed to affect pain perception.

## 5. Conclusion

We reported that the application of an intermittent, high-frequency electrical stimulation protocol for 30 min simultaneously to the thumb and index finger caused an improvement of tactile acuity and of fine motor performance in young adult subjects, but did not alter thresholds of touch and pain. The observation that the improvement of tactile acuity was much larger as compared to previous conditions, where only the index finger had been stimulated, point to synergistic effects as a result of stimulating 2 fingers simultaneously. This assumption is further supported by the improvement of dexterity in fine motor task, which has so far only been observed in elderly individuals characterized by a much poorer baseline performance. These results indicate that the efficacy of repetitive stimulation protocols can be further optimized by selecting appropriate fingers for stimulation, which might be important when such protocols are used for the intervention of impaired subpopulations.
